# Prognostic Impact of Phenotypic and Genetic Features of Pancreatic Malignancies

**DOI:** 10.3390/life15040635

**Published:** 2025-04-11

**Authors:** Mikhail B. Potievskiy, Lidia A. Nekrasova, Ivan V. Korobov, Ekaterina A. Bykova, Ruslan I. Moshurov, Pavel V. Sokolov, Peter A. Shatalov, Natalia A. Falaleeva, Leonid O. Petrov, Vladimir S. Trifanov, Sergey A. Ivanov, Peter V. Shegai, Andrei D. Kaprin

**Affiliations:** 1 FSBI National Medical Research Radiological Centre of the Ministry of Health of the Russian Federation, 249036 Obninsk, Russia; nekrasova.lidia.alexandrovna@gmail.com (L.A.N.); i.korobov99@mail.ru (I.V.K.); bykowayekaterina@yandex.ru (E.A.B.); ruslan4ic93@mail.ru (R.I.M.); sokolov-p-v@yandex.ru (P.V.S.); shatalov.peter@gmail.com (P.A.S.); falaleeva-n@mail.ru (N.A.F.); leonid_petrov@mail.ru (L.O.P.); trifan1975@yandex.ru (V.S.T.); oncourolog@gmail.com (S.A.I.); dr.shegai@mail.ru (P.V.S.); kaprin@mail.ru (A.D.K.); 2 Department of Oncology and Radiology, Institute of Medicine, Peoples’ Friendship University of Russia—RUDN University, 6 Miklukho-Maklaya Street, 117198 Moscow, Russia

**Keywords:** pancreatic neoplasms, genetic heterogeneity, genetic markers, immunohistochemistry, precision oncology, biomarkers

## Abstract

Pancreatic cancer is a tumor with a poor prognosis, and improving its survival outcomes remains a formidable challenge, requiring a multidisciplinary approach that integrates innovative surgical and pharmacological strategies, guided by molecular and genetic insights. The pathomorphological and genetic characteristics of pancreatic cancer, reflected in morphological, immunohistochemical, and serological marker expression, reveal key patterns of tumor genotypic changes during carcinogenesis, aiding in prognostic evaluation and clinical strategy development. The mutational profile of pancreatic tumors is quite heterogeneous and diverse in terms of mutated genes, including in relation to morphological subtypes, but certain patterns have been identified as a result of studies. Pancreatic adenocarcinoma, for instance, is frequently driven by mutations regulating cell division (KRAS). The disease prognosis often depends on the morphological subtype and tumor microenvironment. Neuroendocrine tumors of the pancreas are characterized by a number of pathogenetic features that distinguish them from adenocarcinomas. Thus, neuroendocrine tumors are characterized by mutations of the MENIN protein, which prevents cells from entering the mitosis phase by stimulating the expression of cell cycle regulators. Thus, epithelial and neuroendocrine malignancies of the pancreas differ in immunohistochemical and genetic features, but there are similar mechanisms of pathogenesis, such as BRCA1 and BRCA2 gene mutations, impaired expression of p53 antioncogene, and HIF-2α and mTOR receptor mutations. The predictive impact of serological markers, such as CA 19-9 and CEA, offers insights into tumor metastasis and long-term outcomes, emphasizing the need for personalized therapeutic strategies. Tailoring treatments based on individual molecular profiles holds promise for improving prognosis, as the genetic landscape of pancreatic tumors varies significantly between patients. This underscores the importance of a systematic, patient-specific approach that addresses tumor heterogeneity, resistance mechanisms, and the molecular underpinnings of carcinogenesis.

## 1. Introduction

According to the World Health Organization, pancreatic cancer ranks sixth among tumors in terms of global cancer mortality [1]. Most of the pancreatic tumors have an epithelial origin that carries an exceptionally poor prognosis. Malignant epithelial tumors include ductal adenocarcinoma (PDAC), acinar adenocarcinoma (ACC), and rare pancreatoblastoma, with mixed tumors exhibiting both neuroendocrine and acinar differentiation also identified [2,3,4]. The median survival following surgical treatment ranges from 11.9 to 29.6 months, with 5-year survival rates of 5% to 41% [4,5]. In contrast, neuroendocrine tumors (NENs) exhibit significantly higher survival rates, reaching 93% in the early stages [6,7]. For example, metastatic NEN demonstrates 5-year survival rates ranging from 30% to 80–90% in various studies [8,9].

Despite advances in diagnostic techniques and surgical methods, the overall survival rates for pancreatic malignancies remain poor [10]. The surgical outcomes depend on the disease stage, the chosen surgical method, postoperative complications, and the surgeon’s expertise.

Neoadjuvant chemotherapy reduces the incidence of positive resection margins and improves long-term outcomes. Higher overall and progression-free survival are found with adjuvant treatment and radiation therapy. However, their efficacy is influenced by tumor progression and morphological, genetic, and pathogenetic characteristics [11,12].

Improved long-term outcomes require an understanding of pancreatic cancer pathogenesis. Personalized diagnostic and therapeutic approaches, accounting for prognostic factors such as genetic mutations and treatment sensitivity, are essential for optimizing patient outcomes. Given the distinct pathogenetic features of epithelial and neuroendocrine tumors and the shared genetic mutations across tumor types, evaluating histological subtypes separately is crucial [13,14].

Differences in immunohistochemical and serological marker expression reflect the molecular and genetic heterogeneity of pancreatic tumors. These markers reveal genotype–phenotype associations in the process of carcinogenesis and provide prognostic insights for each tumor type [15,16,17].

This study aims to evaluate the prognostic significance of phenotypic and genetic features of pancreatic malignancies, with a focus on their impact on treatment personalization.

## 2. Materials and Methods

This study provides a comprehensive literature review that was conducted using PubMed and Google Scholar, focusing on studies related to pancreatic cancer. The search terms included “genetic”, “immunohistochemical”, “pancreatic cancer”, “adenocarcinoma”, “neuroendocrine tumors”, “prognostic factors”, “serological markers”, and “genetic features”. The analysis included studies addressing the impact of genetic mutations, immunohistochemical profiles, and serological markers on survival and therapeutic outcomes.

## 3. Prognostic Impact of Genetic and Immunohistochemical Features

Pancreatic tumors, divided into PDACs and neuroendocrine tumors, are additionally classified into several subtypes based on their prognostic impact. PDAC classifications include four immunohistochemical and morphological subtypes, the immune microenvironment, and a classification based on prohibitin expression. In the case of neuroendocrine tumors, the main prognostic impact is associated with immunohistochemical and morphological features.

### 3.1. Histomorphological and Genetic Landscapes of Epithelial Tumors

The development of pancreatic ductal adenocarcinoma (PDAC) is a prolonged process, based on mutation accumulation. Precursor conditions such as pancreatic intraepithelial neoplasia, intraductal papillary neoplasia, and mucinous cystic neoplasia share common developmental mechanisms. However, they differ in histological structure and early genetic changes, ultimately converging into malignancies with similar morphogenetic characteristics [18,19].

A key driver of PDAC is the presence of mutations in the KRAS gene, which encodes a protein involved in the MAP kinase cascade, promoting cell division. These mutations are common across all precursor neoplasia types. Additional mutations, such as in PIK3CA and RNF43, are specific to intraductal papillary and mucinous cystic neoplasia, respectively [19,20,21]. At later stages, tumor suppressor genes, including CDKN2A, TP53, SMAD4, CDK27, and p16, are frequently inactivated, resulting in impaired cell cycle control (G1/S checkpoint) and uncontrolled tumor cell proliferation. Mutations in BRCA genes are also associated with pancreatic cancer development [20,21,22].

Several studies showed the association of various neoplasia types with outcomes. For instance, mucinous cystic neoplasia exhibits higher 5-year survival rates (60%) compared to other tumor types [23]. Additionally, the morphogenetic subtypes of PDAC differ in their immunohistochemical marker expression, which has prognostic significance [24].

Torres C. and Grippo P.J. identified four subtypes of pancreatic adenocarcinoma differing in prognosis and immunohistochemical characteristics. Thus, the squamous subtype is characterized by the expression of ECM, TGFβ, WNT, MYC, and p63 and poor prognosis, with a median survival of 13.3 months. The stem cell subtype is characterized by the expression of PDX1, HNFS, FOXAS, and HES1 and a better prognosis for patients, with a median survival of 23.7 months. The development of the immunogenic subtype is associated with an impaired immune response, characterized by the expression of CTLA4, PD1, PDX1, HNFS, FOXAS, and HES1 and a relatively favorable prognosis. The aberrant endocrine–exocrine tumor subtype is characterized by the expression of markers typical of endocrine and exocrine cells: MIST1, NR5A2, MODY, INS, NEUROD1, NKX2-2 [22,25].

These subtypes do not correspond to the WHO classification, and their immunohistochemical markers differ from those used in routine clinical practice [26]. In addition, for the immunogenic and aberrant endocrine–exocrine subtypes, researchers have not provided data on patient survival, which suggests the need for further investigation of the influence of the immunohistochemical and genetic features of pancreatic adenocarcinoma on treatment outcomes.

### 3.2. Immune Microenvironment and Prognosis of Pancreatic Adenocarcinoma

The study by Tang et al. (2018) evaluated the relationship between the features of the immune response to PDAC and the long-term results (Table 1). The content of immunocompetent cells in peripheral blood and tumor infiltration by different cell types was assessed. According to the results obtained, a higher 5-year survival was observed among patients with a predominance of CD3+ cells in the pancreatic tissue and of monocytes in peripheral blood [23]. According to Nizri E. et al. one of the prognostic factors for survival in patients with metastatic lesions in the lymph nodes is represented by the ratios of T-cells of different types in the lymph nodes [24]. Thus, the predominance of CD8+ cells was associated with a higher 5-year survival. This observation may be related to the tumor suppressor activity of CD8+ cells and suggests that the effectiveness of treatment of cancer patients with lymph node lesions may be related to their immune status [25]. At the same time, there are currently no data on the influence of the amount of cells of this type in the tumor tissue on the long-term results of pancreatic cancer treatment [22].

According to Knudsen et al., the low survival rates of PDAC patients may be associated with specific tumor structure and microenvironment. Thus, infiltration of neoplasms by CTLA4+ T-lymphocytes possessing immunosuppressive activity, as well as by M2-type macrophages, resulted in decreased survival of the patients. This was associated with an “immunosuppressive” structure of the tumor stroma, which, having a low collagen content, contributed to impaired nutrition of the neoplasm and inhibition of tumor-suppressor immunity [22].

Zhang Y. et al., 2019 [27], explored the prognostic impact of immune markers such as PD-1, CD3, and FOX3P. Their findings indicated that high PD-1 expression in the tumor stroma correlated with poorer outcomes, while low levels of PD-1 and FOX3P were associated with improved survival [27]. Conversely, a combination of high PD-1 expression in the stroma and low CD3 expression in the tumor tissue predicted the worst prognosis [27,28].

La Rosa S. et al., 2012 [29], studied the relationship between the expression of BCL10, p53, CK19, CK7, and β-catenin by tumor cells and the survival of patients with ACC. However, their multivariate analysis revealed no significant association between these markers and patient survival, highlighting the need for further research to identify reliable prognostic indicators specific to ACC [29]. Thus, we suggest that the low survival of patients with pancreatic adenocarcinoma is associated with local immunosuppression and hyperexpression of anti-apoptogenic proteins.

### 3.3. Prohibitin

Prohibitin, a transmembrane protein with roles in cellular signaling and mitochondrial function, has emerged as a significant prognostic factor in PDAC. Wang W. et al., 2018 [26], showed that elevated prohibitin expression in the tumor tissue was associated with increased metastatic potential and poorer survival outcomes. The patients with high prohibitin levels exhibited a 60% reduction in median survival compared to those with lower levels. Furthermore, in vitro studies revealed that silencing prohibitin expression reduced tumor growth and invasion, suggesting its potential as a therapeutic target [30,31].

Thus, the interplay between genetic alterations and the immune microenvironment is pivotal in shaping the prognosis of pancreatic cancers. Mutations in key regulatory genes, such as KRAS, TP53, and BRCA1/2, drive PDAC progression, while the tumor immune microenvironment, characterized by immunosuppressive stromal features and variable immune cell infiltration, further modulates patient outcomes. Tumor phenotypic parameters, including prohibitin and immune markers, offer valuable prognostic insights but require further validation in prospective clinical trials. Understanding these molecular and cellular dynamics is critical for the development of personalized therapeutic approaches in pancreatic cancer management.

### 3.4. Genetic Landscape of Neuroendocrine Tumors

Neuroendocrine pancreatic tumors have pathogenetic features different from those of adenocarcinomas (Table 2). For example, NENs are characterized by mutations of the MENIN protein, which prevents cells from entering the mitosis phase by stimulating the expression of cell cycle regulators. However, the role of this factor is ambiguous [32]. MENIN can both increase and decrease the level of expression of various factors that affect cell cycle regulation, which leads to both increased and decreased proliferation in vitro. At the same time, according to the data from clinical studies of patients with metastatic NEN, high expression of MENIN can be associated with a favorable prognosis [33,34].

The activation of telomerase, which leads to mutations in the ATRX and DAXX genes regulating chromatin compaction, plays the key role in the development of neuroendocrine tumors [34,39]. These mutations in combination with increased MENIN expression are associated with the most favorable prognosis for patient survival [32].

### 3.5. Shared Genetic Features Between Epithelial and Neuroendocrine Tumors

**Figure 1:**
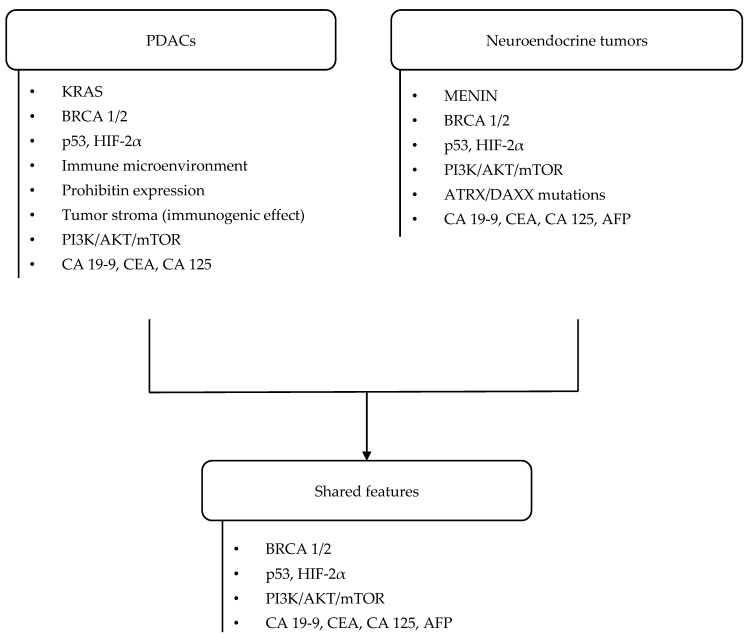
Histomorphological and genetic landscapes of pancreatic tumors.

Having different prognosis and embryological origin, neuroendocrine tumors and pancreatic adenocarcinomas show similar pathogenesis (Figure 1). Both tumor types’ pathogenesis includes BRCA gene mutations, impaired expression of the p53 antioncogene, and HIF-2α and mTOR receptor mutations [40]. mTOR is part of a crucial cell regulatory pathway. Its receptor is a serine/threonine kinase, and it participates in the PI3K/AKT/mTOR signaling pathway, regulates the activity of translation factors, and affects the expression of proteins necessary for cell proliferation [32].

After mTOR activation at the initial stage of this signaling pathway, two complexes, mTORC1 and mTORC2, are activated. The first is able to influence the translation of various hormones and growth factors that regulate the phosphorylation of the 40S subunit of the ribosome and translation elongation factors. mTORC2 activates cascades of reactions mediated by the activation of protein kinase B and protein kinase C, resulting in cytoskeleton rearrangement and increasing the ability of cells to migrate and metastasize [41]. These shared genetic mechanisms underline the complexity of pancreatic tumors and their overlapping, yet unique, molecular profiles.

### 3.6. Immunohistochemical Markers in NENs

Studies evaluating the prognostic value of immunohistochemical markers in pancreatic NENs remain limited (Table 2). For instance, Brunner S. et al., 2015 [38], carried out a comprehensive assessment of factors that influenced the survival of 38 patients with this type of malignant neoplasm. Similar to the data obtained for PDAC, the level of β-catenin expression did not affect patient survival [38]. Similar results were obtained for the content of E-cadherin, cyclin D1, and immunosuppressive interleukin 17 in tumor cells [38], which is able to reduce the activity of T-killer cells in the tumor. This observation suggests that the immune mechanism may be less important in the development of NENs; however, due to the small number of works devoted to this issue, further studies are needed to investigate the influence of tumor-suppressor immunity on the survival of patients with pancreatic NENs.

Smith R.A. et al., 2011 [42], conducted a meta-analysis to evaluate immunohistochemical factors affecting patient survival without regard to the morphological type of neoplasm. The study was statistically analyzed using the Kaplan–Meier algorithm based on data published from 1998 to 2008. According to the authors, the most significant prognostic factor was the expression of VEGF, as an increase in its content in the tumor tissue led to decreased survival. Contradictory results were obtained regarding the prognostic significance of bcl-2, smad4, EGFR, and p53, while a high level of p16 expression was associated with the most favorable prognosis. There was no separation of the data in this meta-analysis by tumor type, and the majority of the observations were related to PDAC. The results obtained suggest that the data presented in the literature on some immunohistochemical markers are inconsistent, which suggests the need for a comprehensive clinical study aimed at identifying prognostic factors for this type of tumor in different groups of patients, taking into account age, stage, and tumor morphology [42].

Data on the influence of immunohistochemical characteristics of pancreatic NEN are presented in the review paper by Capruso G. et al., 2015 [32]. Thus, according to the authors, the level of expression of vascular growth factors does not affect the survival rate of patients with neuroendocrine tumors. At the same time, the study presented conclusions about the contradictions with other studies on the significance of VEGF, VEGFR, and PDGF [34].

Increased EGFR expression may also be a manifestation of the pathological activity of the PI3K/AKT/mTOR and MAPK signaling pathways [34], which may be associated with a prognosis of lower survival for patients with pancreatic NEN [37].

The proliferation marker Ki-67 has been widely studied as a prognostic tool for NEN. High Ki-67 expression is associated with poorly differentiated tumors and increased metastatic potential. This observation is supported by the World Health Organization (WHO) classification, which uses Ki-67 levels to stratify NENs into prognostic categories. Elevated Ki-67 levels are consistently linked to unfavorable outcomes and higher risks of disease progression [35,36,40].

Thus, the pathogenesis of pancreatic NEN is poorly understood, and the data presented in the current literature are largely contradictory. However, the pathogenesis of this tumor type is largely associated with the disruption of cell cycle regulation (G1/S checkpoint), similar to the pathogenesis of pancreatic adenocarcinoma [43,44,45].

## 4. Prognostic Impact of Serological Markers

Serological tumor markers play an important role in diagnosing and monitoring digestive system cancers, including pancreatic cancer [13,46]. Elevated levels of these markers in blood plasma reflect phenotypic changes in tumor cells, making them valuable tools for both diagnostic and prognostic purposes [47].

CA 19-9 is one of the most widely used markers in clinical practice. This glycoprotein, a component of the glycocalyx, becomes overexpressed in pancreatic tumors due to accumulating mutations during carcinogenesis [48,49]. Elevated CA 19-9 levels are strongly associated with advanced disease and poor prognosis. Numerous studies have confirmed its reliability as a diagnostic marker, with patients exhibiting CA 19-9 levels below 37 U/mL generally demonstrating better survival outcomes [50].

Carcinoembryonic antigen (CEA) is another key marker for gastrointestinal malignancies. As a glycoprotein facilitating cell adhesion during embryonic development, its expression is typically low in normal tissues but increases significantly in malignant transformation. Elevated CEA levels are associated with pancreatic cancer and predict worse outcomes. Patients with CEA levels below 5 ng/mL generally achieve the most favorable survival rates [50,51].

CA 125, a high-molecular-weight glycoprotein, is utilized in the diagnosis of various cancers. Elevated CA 125 levels are often linked to pancreatic cancer progression and metastasis, with high levels generally indicating a poor prognosis. Interestingly, a study by Balachandran V.P. et al. identified a paradoxical association between high CA 125 expression in tumor cells and improved survival of pancreatic adenocarcinoma patients, which the authors attributed to enhanced recognition of these cells by CD8+ immune cells [52].

Similarly to PDACs, neuroendocrine neoplasms are characterized by increased expression of multiple markers (Figure 1), including CA 19-9, CEA, CA 125, and α-fetoprotein (AFP). Chen L. et al. reported that elevated CA 125 levels in patients with pancreatic NEN are often associated with distant metastases and a poor prognosis. Moreover, the simultaneous elevation of two or more markers correlates with significantly reduced survival rates [53].

The overexpression of tumor markers is often driven by mutations in multiple genes, reflecting the cumulative genetic instability of tumor cells. For example, CA 19-9 synthesis is regulated by several genes on chromosome 19. According to He M. et al., mutations in a single gene can increase CA 19-9 expression by an average of 2.5 U/mL. Similar associations have been observed for CEA and AFP, with specific regions on chromosome 19 implicated in the co-elevation of both CA 19-9 and CEA levels [54].

These findings suggest that the accumulation of mutations during carcinogenesis drives the increased expression of serological markers, further exacerbating genetic instability, enhancing malignancy, and worsening prognosis. Consequently, the plasma levels of tumor markers are indirect indicators of carcinogenesis, tumor evolution, and therapeutic response, as evidenced by clinical studies [55,56,57].

In a study by Lee K.J. et al., the prognostic utility of CA 19-9 and CEA was compared in non-resectable pancreatic adenocarcinoma [58]. The results showed that lower CEA levels were associated with slower disease progression and improved survival, whereas the CA 19-9 levels did not show significant prognostic differences in this patient group [50,58].

In NEN, the levels of serological markers indicative of neuroendocrine differentiation, such as chromogranin A and synaptophysin, may be elevated [59,60,61]. Elevated levels of these markers, particularly when combined with high Ki-67 staining in immunohistochemistry, are associated with poor prognosis. However, low-grade tumors may not exhibit detectable levels of these markers, which complicates their use in diagnosis and prognosis [60,62,63,64].

Zhuge X. et al. reported that elevated CA 19-9 and CEA levels are typically observed in low-grade neuroendocrine tumors, which can aid in differentiating between epithelial and neuroendocrine pancreatic tumors. Increased levels of these markers, in the presence of neuroendocrine differentiation, may indicate an epithelial component within the tumor, which is associated with worse outcomes [65,66].

## 5. Prospects for the Development of Individual Therapeutic and Diagnostic Approaches for Pancreatic Cancer Treatment

The development of pancreatic tumors is associated with the accumulation of mutations, often driven by mitotic abnormalities. Many tumor cells exhibit polyploidy, leading to increased chromosomal mutations and genetic instability. This heightened genetic instability further drives genomic alterations in tumor cells, contributing to mutation accumulation and phenotypic changes [67,68,69]. This process, known as tumor microevolution, is described by Notta F. et al., who identified that key mutations often arise during the diploid stage, with polyploidization accelerating these pathological changes [56,70].

These insights underscore the urgent need for a paradigm shift in pancreatic cancer diagnosis and treatment. Early detection is particularly critical, as early-stage pancreatic tumors can rapidly acquire metastatic potential, increasing the risk of disease spread and recurrence [56,59,67,71].

Leveraging insights into the stage of tumor development can provide valuable information about tumor progression, metastatic risk, overall prognosis, and optimal treatment strategies to improve patient survival and quality of life [16].

Individual tumor characteristics, such as alterations in immunohistochemical marker expression, have a strong potential to predict drug resistance and poor outcomes. Additionally, routine monitoring of serological markers like CA 19-9, CA 125, reticuloendothelial antigen, α-fetoprotein, and chromogranin A before and during treatment offers critical insights for diagnostic, prognostic, and therapeutic planning [47,71].

The understanding of pancreatic cancer subtypes allows for achieving optimal treatment effectiveness. Specific mutations make possible the administration of targeted drugs. For example, the PARP inhibitor olaparib is effective in patients with BRCA 1,2 mutations, while enretinib is effective in ROS-1- mutated patients, and sotorasib and afatinib are effective in KRAS-mutated patients [72]. Other functional mutations indicate resistance to specific drugs. According to Neoptolemos J.P. et al., 2023, CYP3A5 mutation is associated with increased irinotecan metabolization and poorer outcomes of FOFORINOX chemotherapy [73]. At the same time, molecular and genetic tumor subtyping is not fully included in the routine examination of patients with pancreatic cancer [72].

The development of personalized treatment protocols for pancreatic cancer patients requires a comprehensive approach that should consider the molecular and genetic underpinnings of the disease, the mechanisms of action of targeted therapies, and possible drug resistance [74,75]. These suggestions uncover various possibilities for future multicenter prospective studies.

## 6. Conclusions

Epithelial and neuroendocrine pancreatic tumors, while distinct in their morphological and genetic profiles, share common pathogenic mechanisms, including BRCA1 and BRCA2 gene mutations, impaired p53 function, and HIF-2α and mTOR receptor alterations. Pancreatic tumors exhibit significant morphogenetic heterogeneity, characterized by diverse marker expression and histological features, which impacts overall patient survival and treatment response. PDAC classifications include morphological, immunological/microenvironmental, and “prohibitin” approaches. While the development of pancreatic tumors follows common patterns, genetic, morphological, and microenvironmental variations influence their prognosis. A poor prognosis is usually connected with the activation of proliferative pathways and the expression of associated markers. Therefore, a personalized approach, based on tumor genomics and morphology, is essential to optimize treatment outcomes.

## Figures and Tables

**Table 1:** Prognostic impact of genetic and phenotypic features in pancreatic adenocarcinomas.

Feature	Pathogenesis	Mechanism of Action in Pancreatic Cancer Pathogenesis	Prognostic Impact	References
CD3+ cells	T-cell population	Represents total T-cell infiltration; contributes to anti-tumor immune response via activation of cytotoxic and helper T-cells	Associated with higher 5-year survival	Tang et al., 2018 [23]
CD8+ cells	Cytotoxic T-cells	Directly kill tumor cells through perforin and granzyme release, key players in adaptive anti-tumor immunity	Linked to improved prognosis and tumor suppression	Nizri et al., 2019 [24]
CTLA4+ T-lymphocytes	Immune suppression	CTLA-4 inhibits T-cell activation by outcompeting CD28 for B7 ligands on antigen-presenting cells, reducing immune attack on the tumor	Infiltration correlates with decreased survival	Knudsen et al., 2020 [21]
Prohibitin	Transmembrane protein	Involved in mitochondrial function and cell cycle regulation; overexpression may promote tumor cell survival and metastatic potential	High levels linked to increased metastasis risk and unfavorable prognosis	Wang et al., 2018 [26]
PD-1, FOX3P expression	Immune regulation	PD-1 inhibits T-cell function upon ligand binding (PD-L1), leading to immune evasion; FOXP3 marks regulatory T-cells that suppress immunity	High PD-1 and low CD3 expression correlates with poor prognosis	Zhang et al., 2019 [27]
Tumor stroma collagen content	Structural component	Collagen provides a scaffold for immune cell migration; low content may hinder immune cell infiltration and impair anti-tumor immunity	Low collagen content is linked to impaired tumor-suppressor immunity and poorer survival	Knudsen et al., 2020 [21]

**Table 2:** Prognostic significance of genetic and phenotypic features of neuroendocrine pancreatic tumors.

Feature	Pathogenesis	Mechanism of Action in Pancreatic Cancer Pathogenesis	Prognostic Significance	References
MENIN expression	Regulates cell cycle and prevents mitosis	Acts as a tumor suppressor by inhibiting transcription of pro-proliferative genes; loss may lead to endocrine tumor development	High expression associated with favorable prognosis in metastatic neuroendocrine tumors	Capurso et al., 2015 [32]; Majer et al, 2024 [33]
ATRX/DAXX mutations	Chromatin remodeling	Loss of function leads to alternative lengthening of telomeres (ALT), contributing to genomic instability and tumor progression	Associated with favorable prognosis in combination with high MENIN expression	van’t Veld et al, 2025 [35]
Ki-67 expression	Cell proliferation marker	Reflects the fraction of actively dividing cells; high expression indicates aggressive tumor behavior	High levels are associated with poor prognosis in poorly differentiated tumors	Sorbye et al, 2013 [36]
VEGF expression	Vascular endothelial growth factor	Promotes angiogenesis and vascular permeability, enhancing tumor growth and potential for metastasis	Not significantly associated with survival in neuroendocrine tumors	Capruso et al., 2015 [32]
EGFR expression	Epidermal growth factor receptor	Activates PI3K/AKT/mTOR and MAPK pathways, leading to cell proliferation, survival, and migration	Associated with poor prognosis due to pathway activation	Stanciu S. et al., 2022 [37]
β-catenin, CK19, CK7, p53	Tumor cell markers	β-catenin: Wnt signaling and cell adhesion; CK19/CK7: epithelial origin markers; p53: genomic stability via DNA repair and apoptosis (often mutated)	No significant impact on patient survival	La Rosa et al., 2012 [29]; Brunner et al., 2015 [38]

## Data Availability

No new data were created or analyzed in this study.
